# Rousing a Latent Defense Mechanism to Fight HIV

**DOI:** 10.1371/journal.pbio.1000093

**Published:** 2009-04-28

**Authors:** Caitlin Sedwick

Despite decades of effort, medical science hasn't yet succeeded in preventing or curing infections by human immunodeficiency virus (HIV), the virus that causes AIDS. The virus's rapid mutation rate has so far prevented the creation of a vaccine, leaving the medical community to fight individual infections with drugs that inhibit viral replication. These drugs, while effective, are not a cure. Additionally, they're costly, have serious side-effects, and must be administered for life. So, the search continues for other approaches to help prevent new infections, and to treat or cure existing ones. In this issue of *PLoS Biology*, Nitya Venkataraman, Alexander Cole, and colleagues describe one of these new approaches: awakening a latent defense mechanism in human cells.

Venkataraman et al. focused their work on retrocyclin, a member of the defensin family of proteins. Defensins are made by all primates and have potent anti-microbial activity. Retrocyclin was recently shown to strongly inhibit HIV entry into human cells by blocking the interaction of viral proteins with their cellular receptors. Unfortunately, although the retrocyclin protein is found in Old World monkeys and orangutans, it is not present in humans (or gorillas and chimps), even though we have the retrocyclin gene.

That's because the human retrocyclin gene contains a mutation, known as a “nonsense mutation,” that prevents the production of the encoded protein. Nonsense mutations are a common cause of many human hereditary diseases, including cystic fibrosis, some cancers, and muscular dystrophies. They work by disrupting the genes that contain the instructions for making specific proteins. Information in genes, encoded in nucleotide triplets called “codons,” is preserved as the DNA sequence is transcribed into the RNA molecules that carry it to the cell's protein factory, the ribosome. Ribosomes read the order of the nucleotides within codons to determine which amino acids (protein building blocks) should be incorporated into a protein, and in what order. When a ribosome encounters “stop codons,” it dutifully stops adding amino acids to a protein. Nonsense mutations change an amino acid–encoding codon into a premature stop codon, resulting in a truncated protein—or no protein at all—being made.

No one knows how or why humans acquired a nonsense mutation in the retrocyclin gene. But, since monkey retrocyclin has the ability to block HIV infection, Venkataraman et al. decided it would be worth investigating whether the human version of the protein is similarly useful. Almost immediately, they encountered a potential problem: even if modern human cells had a functional copy of the gene available, they may not be able to make retrocyclin protein. That's because the monkey retrocyclin protein has an unusual 3D structure that is unique. The production of retrocyclin protein might require some specialized mechanism, which could be absent in humans.

To determine whether human cells have retained the capacity to make retrocyclin protein, Venkataraman et al. corrected the premature stop codon mutation in a copy of the human retrocyclin gene. Next, they inserted the corrected gene into human promyelocytic cells, and looked to see if protein was produced from the gene. They found that cells harboring the corrected gene could make a protein similar to the monkey version of retrocyclin. But could human retrocyclin block HIV infection? Indeed, extracts made from cells containing the corrected gene could reduce HIV growth, and so could the retrocyclin protein purified from these extracts. Collectively, these results suggest that human cells have a potentially important—but latent—mechanism to protect against HIV.

If retrocyclin could be restored in humans, it might make a difference in the fight against HIV. The authors therefore wondered if it would be possible to make human cells ignore the premature stop codon in the retrocyclin gene. To accomplish this, they turned to aminoglycosides, compounds known to make human cells ignore premature stop codons. Aminoglycosides are normally used to fight off bacterial infections; they work by binding tightly to bacterial ribosomes, blocking their protein-making capacity. These compounds can also bind to human ribosomes, but they do so much more weakly. Therefore, they don't block protein creation in human cells but cause ribosomes to make occasional errors—like missing stop codons. The authors found that treating human cells with aminoglycosides allowed the cells to make retrocyclin at sufficiently high levels to inhibit infection by HIV. Importantly, the authors showed that antibodies specific to retrocyclin could destroy the HIV-blocking activity of aminoglycoside-treated cells, indicating that the anti-HIV activity of aminoglycoside-treated cells mediates their new ability to produce retrocyclin.

Might aminoglycosides, or drugs like them, be useful in rallying this ancient defense mechanism to battle against HIV? Much more work would be needed to demonstrate the safety and effectiveness of this approach for stimulating retrocyclin production, and to investigate whether this could actually protect people from HIV. But these findings represent a promising step in that direction.

